# Supplementary material for: Boys don’t cry (or kiss or dance): A computational linguistic lens into gendered actions in film
Source: PLoS One. 2022 Dec 21;17(12):e0278604. doi: 10.1371/journal.pone.0278604 (PMC9770346; doi:10.1371/journal.pone.0278604)
Supplement: S1 Table — Complete table of performance results for the SRL systems. Legend: Oracle action denotes models with no automatic action identification. Uncased / Cased refers to the type of pre-trained BERT model used. +fine-tune notes models that were fine-tuned using the manually labeled action description dataset. For LSTM and GRU, size of hidden dimension is given by the number in parenthesis. Best performance highlighted in bold. (PDF) [file pone.0278604.s001.pdf]

S1 Table. Machine Learning Model Performance Complete table of performance results for the SRL systems. Legend: Oracle action denotes models with no automatic action identification. Uncased / Cased refers to the type of pre-trained BERT model used. +fine-tune notes models that were fine-tuned using the manually labeled action description dataset. For LSTM and GRU, size of hidden dimension is given by the number in parenthesis. Best performance highlighted in bold.

Model	Accuracy	micro-F1	Action F1	Agent Precision	Agent Recall	Agent F1	Patient Precision	Patient Recall	Patient F1
BASELINES									
Shi et al. [49] (oracle action)	78.20%	84.10%	–	95.10%	63.42%	76.10%	41.30%	38.33%	39.76%
Shi et al. [49]	51.90%	63.00%	69.70%	91.55%	54.78%	68.55%	29.44%	34.87%	31.93%
Gardner et al. [57] (oracle action)	60.65%	74.18%	–	90.86%	31.99%	47.31%	72.46%	28.82%	41.24%
Gardner et al. [57]	41.80%	53.05%	69.23%	71.63%	27.85%	40.11%	37.44%	23.63%	28.98%
Sap et al. [29] (oracle action)	64.54%	77.82%	–	93.82%	54.41%	68.88%	94.62%	35.45%	51.57%
Sap et al. [29]	48.60%	63.91%	69.14%	86.15%	49.72%	63.05%	85.19%	26.51%	40.44%
BERT [34] + LSTM									
Uncased + LSTM (50)	38.80%	54.20%	34.68%	87.72%	55.32%	67.85%	0.00%	0.00%	0.00%
Uncased + LSTM (100)	32.40%	47.60%	22.24%	87.81%	55.78%	68.23%	0.00%	0.00%	0.00%
Uncased + LSTM (300)	31.50%	45.50%	17.82%	86.03%	58.02%	69.30%	0.00%	0.00%	0.00%
Cased + LSTM (50)	63.70%	70.30%	76.64%	86.72%	60.91%	71.56%	43.90%	31.58%	36.73%
Cased + LSTM (100)	69.90%	70.90%	77.63%	86.80%	60.73%	71.46%	33.33%	0.88%	1.71%
Cased + LSTM (300)	68.90%	74.20%	81.88%	86.84%	60.91%	71.60%	47.59%	63.45%	54.39%
Cased + LSTM (50) + fine-tune	89.00%	90.70%	96.91%	89.40%	88.15%	88.77%	73.94%	66.37%	69.95%
Cased + LSTM (100) + fine-tune	89.00%	90.90%	96.63%	90.14%	88.71%	89.42%	55.75%	65.20%	60.11%
Cased + LSTM (300) + fine-tune	89.30%	91.20%	96.84%	88.90%	90.39%	89.64%	82.42%	61.70%	70.57%
Cased + LSTM (500) + fine-tune	88.80%	90.80%	96.47%	89.64%	88.81%	89.22%	77.51%	65.50%	71.00%
BERT [34] + GRU									
Cased + GRU (50)	67.80%	73.40%	87.11%	86.84%	60.91%	71.60%	45.55%	50.88%	48.07%
Cased + GRU (100)	67.10%	72.70%	79.60%	86.85%	61.01%	71.67%	45.63%	61.11%	52.25%
Cased + GRU (300)	69.30%	74.20%	82.04%	86.79%	61.29%	71.84%	46.60%	66.08%	54.66%
Cased + GRU (50) + fine-tune	**90.20%**	**91.30%**	96.80%	89.82%	**89.74%**	**89.78%**	74.10%	**71.93%**	**73.00%**
Cased + GRU (100) + fine-tune	88.30%	90.90%	96.21%	89.92%	89.09%	89.50%	**82.06%**	62.87%	71.19%
Cased + GRU (300) + fine-tune	89.20%	91.00%	**96.85%**	**90.40%**	87.87%	89.12%	75.90%	68.13%	71.80%
