# Supplementary material for: Boys don’t cry (or kiss or dance): A computational linguistic lens into gendered actions in film
Source: PLoS One. 2022 Dec 21;17(12):e0278604. doi: 10.1371/journal.pone.0278604 (PMC9770346; doi:10.1371/journal.pone.0278604)
Supplement: S2 Table — Regression model results for agent’s actions. We test the significance of the coefficients through Z-test, and correct for multiple comparisons using the Holm-Bonferroni method. Table shows only significant coefficients with adjusted-p < 0.05. Rows are ordered by the magnitude of their coefficient (β). The direction of the relationship is determined by the sign of the coefficient, with positive coefficients corresponding to actions which are more likely portrayed by a male character. Likewise, negative coefficients present actions that are less likely to be portrayed by male characters. Manually identified errors are color coded (blush—errors due to parsing and lemmatization; gray—errors due to SRL). (PDF) [file pone.0278604.s002.pdf]

S2 Table. Results for Study 1: Agent-only Regression model results for agent's actions. We test the significance of the coefficients through Z-test, and correct for multiple comparisons using the Holm-Bonferroni method. Table shows only significant coefficients with adjusted-$p < 0.05$. Rows are ordered by the magnitude of their coefficient (β). The direction of the relationship is determined by the sign of the coefficient, with positive coefficients corresponding to actions which are more likely portrayed by a male character. Likewise, negative coefficients present actions that are less likely to be portrayed by male characters. Manually identified errors are color coded (blush - errors due to parsing and lemmatization; gray - errors due to SRL).

Study 1: Actions were the agent is more likely to be male

Action	Estimate (β)	Std. Error	Z
sigh start	3.26	1.03	3.16
asleep	2.79	0.75	3.70
perturb	2.58	0.90	2.85
stare fascinate[d]	2.28	0.81	2.83
stride	0.87	0.28	3.09

Study 1: Actions were the agent is less likely to be male

Action	Estimate (β)	Std. Error	Z
cry	-1.01	0.27	-3.81
giggle	-1.03	0.33	-3.17
looks	-1.23	0.40	-3.07
flex	-1.36	0.47	-2.89
stream	-1.39	0.31	-4.45
track	-1.39	0.32	-4.31
sob	-1.47	0.31	-4.71
snuggle	-1.49	0.48	-3.10
quiver	-1.49	0.51	-2.92
review	-1.52	0.50	-3.03
will do	-1.65	0.58	-2.87
s quiet	-1.78	0.63	-2.83
pans	-1.83	0.47	-3.93
ricochet	-1.85	0.63	-2.94
belch	-2.01	0.68	-2.96
outstretche	-2.12	0.68	-3.14
cluck	-2.26	0.60	-3.79
plan	-2.36	0.65	-3.61
unholster	-2.40	0.81	-2.98
start gather	-2.47	0.77	-3.20
ladle	-2.72	0.87	-3.11
turn nod	-2.73	0.95	-2.88
patch	-2.76	0.81	-3.40
disclose	-2.77	0.82	-3.39
start back	-2.78	0.81	-3.43
undaunte	-2.79	0.84	-3.31
emblazon	-2.82	0.83	-3.40
would say	-2.82	0.95	-2.97
would look	-2.83	0.86	-3.31

Study 1: Actions were the agent is less likely to be male

Action	Estimate (β)	Std. Error	Z
can muster	-2.90	0.82	-3.52
blank	-2.96	0.75	-3.93
finish cut	-3.02	1.03	-2.92
swap	-3.07	1.09	-2.81
fast	-3.11	1.03	-3.01
pucker	-3.16	0.75	-4.21
compete	-3.23	0.81	-4.00
hightail	-3.23	1.13	-2.87
fires miss	-3.26	1.15	-2.84
sit write	-3.27	1.11	-2.94
look concern	-3.27	1.11	-2.94
turn glance	-3.31	1.15	-2.88
lever	-3.32	1.09	-3.04
get rid	-3.40	1.10	-3.09
start rub	-3.43	0.90	-3.80
blossom	-3.43	1.03	-3.33
stare horrify	-3.44	0.95	-3.63
powder	-3.46	1.03	-3.35
disrobe	-3.48	1.18	-2.94
portray	-3.49	1.18	-2.95
affront	-3.53	1.25	-2.82
nod shake	-3.61	1.25	-2.88
notice turn	-3.63	1.18	-3.07
game	-3.63	1.25	-2.90
begin do	-3.64	1.18	-3.08
enter toss	-3.70	1.03	-3.58
jar	-3.70	1.08	-3.41
effect	-3.71	1.25	-2.97
look baffle	-3.72	1.11	-3.35
exceed	-3.78	1.25	-3.02
choke gasping	-3.79	1.25	-3.03
orbit	-3.84	0.95	-4.05
encrust	-3.87	1.25	-3.09
can offer	-3.88	1.25	-3.10
stare wait	-3.90	1.25	-3.12
stop follow	-3.91	1.25	-3.13
stop spin	-3.97	1.18	-3.35
rivite	-4.06	1.25	-3.24
nod sip	-4.07	1.18	-3.44
start whip	-4.30	1.25	-3.44
adapt	-4.32	0.64	-6.77
reprimand	-4.38	1.25	-3.50
gut punch	-4.91	1.25	-3.92
