# Supplementary material for: Boys don’t cry (or kiss or dance): A computational linguistic lens into gendered actions in film
Source: PLoS One. 2022 Dec 21;17(12):e0278604. doi: 10.1371/journal.pone.0278604 (PMC9770346; doi:10.1371/journal.pone.0278604)
Supplement: S3 Table — Regression model results for actions done to patients. We test the significance of the coefficients through Z-test, and correct for multiple comparisons using the Holm-Bonferroni method. Table shows only significant coefficients with adjusted-p < 0.05. Rows are ordered by the magnitude of the coefficients (β). The direction of the relationship is determined by the sign of the coefficient, with positive coefficients corresponding to actions more likely done towards male characters. Manually identified errors are color coded (blush—errors due to parsing and lemmatization; gray—errors due to SRL). (PDF) [file pone.0278604.s003.pdf]

S3 Table. Results for Study 2: Patient-only Regression model results for actions done to patients. We test the significance of the coefficients through Z-test, and correct for multiple comparisons using the Holm-Bonferroni method. Table shows only significant coefficients with adjusted-$p < 0.05$. Rows are ordered by the magnitude of the coefficients (β). The direction of the relationship is determined by the sign of the coefficient, with positive coefficients corresponding to actions more likely done towards male characters. Manually identified errors are color coded (blush - errors due to parsing and lemmatization; gray - errors due to SRL).

Study 2: Actions were the patient is more likely to be male

Action	Estimate (β)	Std. Error	Z
kiss	3.63	1.61	2.26
corrode	3.54	1.44	2.45
begin singe	3.38	1.44	2.34
headline	3.26	1.44	2.26
shoot	3.19	1.45	2.21
retrace	3.12	1.44	2.16
volunteer	3.12	1.44	2.16
hear	3.04	1.44	2.10
infect	2.94	1.44	2.04
grimace	2.86	1.36	2.11
cavort	2.78	1.39	2.01
brew	2.74	1.39	1.98
unsnap	2.70	1.26	2.15
lob	2.31	1.12	2.06

Study 2: Actions were the patient is less likely to be male

Action	Estimate (β)	Std. Error	Z
kidnap	-2.21	1.12	-1.97
boot	-2.26	1.08	-2.09
rot	-2.30	0.97	-2.37
lure	-2.41	1.08	-2.23
gutte	-2.45	1.19	-2.06
leafs	-2.51	1.08	-2.33
strum	-2.53	1.19	-2.13
hassle	-2.60	1.26	-2.07
compress	-2.61	1.16	-2.26
will pray	-2.65	1.19	-2.22
proposition	-2.69	1.19	-2.26
begin talk	-2.70	1.35	-1.99
rise go	-2.71	1.34	-2.02
gawk	-2.76	1.12	-2.47
lie scatter	-2.78	1.39	-2.00
pity	-2.81	1.39	-2.03
look	-2.88	1.36	-2.12
drug	-2.89	1.44	-2.00
start leak	-2.89	1.44	-2.00
endow	-2.92	1.44	-2.02
fence	-2.93	1.32	-2.23

cherish	-2.98	1.44	-2.07
be pull	-3.00	1.44	-2.08
stand	-3.03	1.45	-2.09
closes	-3.05	1.39	-2.20
nake	-3.09	1.44	-2.14
comply	-3.13	1.39	-2.26
extricate	-3.18	1.61	-1.98
expel	-3.19	1.39	-2.30
doff	-3.20	1.61	-1.99
package	-3.24	1.44	-2.25
seem aware	-3.29	1.44	-2.28
stop	-3.36	1.44	-2.33
see	-3.38	1.44	-2.34
come	-3.39	1.44	-2.35
lilt	-3.43	1.61	-2.13
pass touch	-3.45	1.61	-2.15
constrict	-3.47	1.45	-2.40
ad libbe	-3.48	1.61	-2.17
dole	-3.48	1.61	-2.17
remember	-3.60	1.61	-2.24
dazed	-3.65	1.61	-2.27
fall	-3.65	1.61	-2.27
plot	-3.71	1.45	-2.56
rivite	-3.73	1.61	-2.32
seat	-3.85	1.61	-2.39
expressionless look	-4.13	1.61	-2.56
