# Supplementary material for: Boys don’t cry (or kiss or dance): A computational linguistic lens into gendered actions in film
Source: PLoS One. 2022 Dec 21;17(12):e0278604. doi: 10.1371/journal.pone.0278604 (PMC9770346; doi:10.1371/journal.pone.0278604)
Supplement: S4 Table — Regression model results for the agent–patient interactions. Group encodes gender dynamics (e.g., M→M identify actions done by male characters towards other male characters). We test the significance of the coefficients through Z-test, and correct for multiple comparisons using the Holm-Bonferroni method. Table shows only significant coefficients with adjusted-p < 0.05. Rows are ordered by the magnitude of the coefficients (β). Direction of the relationship is given by the sign and magnitude of β with positive values indicating actions more likely portrayed by that group of characters. Manually identified errors are color coded: blush for errors down-streamed from an outside the SRL system (e.g., parsing, lemmatization), and gray for errors due to mislabels coming from our SRL system. (PDF) [file pone.0278604.s004.pdf]

**S4 Table. Results for Study 3: Agent & Patient** Regression model results for the agent–patient interactions. Group encodes gender dynamics (e.g., M→M identify actions done by male characters towards other male characters). We test the significance of the coefficients through Z-test, and correct for multiple comparisons using the Holm-Bonferroni method. Table shows only significant coefficients with adjusted-$p < 0.05$. Rows are ordered by the magnitude of the coefficients ($\beta$). Direction of the relationship is given by the sign and magnitude of $\beta$ with positive values indicating actions more likely portrayed by that group of characters. Manually identified errors are color coded: blush for errors down-streamed from an outside the SRL system (e.g., parsing, lemmatization), and gray for errors due to mislabels coming from our SRL system.

Study 3: Actions more likely done by a male character to another male character

| Group | Action | Estimate | Std. Error | Z |
|---|---|---|---|---|
| M→M | kiss | -3.18 | 0.75 | -4.27 |
| M→M | dance | -2.91 | 0.91 | -3.19 |
| M→M | wrap | -2.91 | 0.77 | -3.80 |
| M→M | hug | -2.78 | 0.75 | -3.70 |
| M→M | hesitate | -2.71 | 0.88 | -3.10 |
| M→M | cover | -2.59 | 0.75 | -3.45 |
| M→M | scream | -2.59 | 0.75 | -3.46 |
| M→M | remove | -2.49 | 0.78 | -3.20 |
| M→M | laugh | -2.46 | 0.74 | -3.34 |
| M→M | eat | -2.44 | 0.85 | -2.88 |
| M→M | face | -2.43 | 0.82 | -2.95 |
| M→M | wear | -2.35 | 0.73 | -3.20 |
| M→M | begin | -2.34 | 0.74 | -3.17 |
| M→M | close | -2.34 | 0.73 | -3.19 |
| M→M | hurry | -2.31 | 0.78 | -2.95 |
| M→M | lie | -2.30 | 0.75 | -3.08 |
| M→M | set | -2.29 | 0.75 | -3.03 |
| M→M | let | -2.27 | 0.73 | -3.10 |
| M→M | dress | -2.22 | 0.75 | -2.95 |
| M→M | kneel | -2.21 | 0.76 | -2.92 |
| M→M | find | -2.21 | 0.73 | -3.02 |
| M→M | hear | -2.20 | 0.73 | -3.01 |
| M→M | seem | -2.19 | 0.74 | -2.96 |
| M→M | smile | -2.19 | 0.73 | -3.01 |
| M→M | roll | -2.17 | 0.74 | -2.92 |
| M→M | hold | -2.13 | 0.73 | -2.94 |
| M→M | cross | -2.12 | 0.74 | -2.87 |
| M→M | start | -2.11 | 0.73 | -2.90 |
| M→M | move [towards] | -2.08 | 0.73 | -2.86 |
| M→M | continue | -2.07 | 0.74 | -2.82 |
