# Supplementary material for: Boys don’t cry (or kiss or dance): A computational linguistic lens into gendered actions in film
Source: PLoS One. 2022 Dec 21;17(12):e0278604. doi: 10.1371/journal.pone.0278604 (PMC9770346; doi:10.1371/journal.pone.0278604)
Supplement: S5 Table — Regression model results for the agent–patient interactions controlling for year of production. Group encodes gender dynamics (e.g., M→M identify actions done by male characters towards other male characters). A star (*) is used as short-hand for either group (e.g., *→F labels actions where the patient is Female and the agent’s gender can be any value). We test the significance of the coefficients through Z-test, and correct for multiple comparisons using the Holm-Bonferroni method. Table shows only significant coefficients with adjusted-p < 0.05. Rows are ordered by the magnitude of the coefficient (β). The direction of the relationship is given by the coefficient’s sign, with positive coefficients corresponding to actions more likely portrayed by their encoding group. Manually identified errors are color coded gray for errors due to mislabels coming from our SRL system. (PDF) [file pone.0278604.s005.pdf]

S5 Table. Results for Study 4: Agent & Patient + Time Regression model results for the agent–patient interactions controlling for year of production. Group encodes gender dynamics (e.g., M→M identify actions done by male characters towards other male characters). A star (*) is used as short-hand for either group (e.g., *→F labels actions where the patient is Female and the agent's gender can be any value). We test the significance of the coefficients through Z-test, and correct for multiple comparisons using the Holm-Bonferroni method. Table shows only significant coefficients with adjusted-$p < 0.05$. Rows are ordered by the magnitude of the coefficient (β). The direction of the relationship is given by the coefficient's sign, with positive coefficients corresponding to actions more likely portrayed by their encoding group. Manually identified errors are color coded gray for errors due to mislabels coming from our SRL system.

Study 4: Actions more likely done by a gendered agent through the decades

Group	Action	Estimate	Std. Error	Z
M→*	look	73.62	12.68	5.81
M→*	turn	34.24	12.68	2.70
M→*	take	33.16	12.68	2.62
M→*	see	32.36	12.68	2.55
M→*	pull	26.09	12.68	2.06
F→*	adapt	-115.78	16.33	-7.09

Study 4: Actions more likely done to a gendered patient through the decades

Group	Action	Estimate	Std. Error	Z
*→M	see	36.66	5.85	6.27
*→M	turn [towards]	20.16	5.85	3.45
*→M	stand	16.23	5.85	2.78
*→M	take	15.51	5.85	2.65
*→M	pull	14.91	5.85	2.55
*→M	walk [to]	14.34	5.85	2.45
*→M	sit	12.77	5.85	2.18
*→M	go	12.15	5.85	2.08
*→F	come	-13.49	5.85	2.31
*→F	watch	-13.87	5.85	2.37
*→F	look	-50.26	5.84	8.59

Study 4: Actions more likely done by a gendered interaction through the decades

Group	Action	Estimate	Std. Error	Z
F→M	reach	10.05	3.96	2.53
F→N	open	25.35	8.47	2.99
M→M	call meeting	8.83	4.39	2.01
M→M	get pissed	6.45	2.69	2.40
N→M	see	13.40	4.48	2.99
N→M	dance	11.69	5.00	2.34
M→M	dress intelligent	9.74	2.70	3.61
