# Supplementary material for: Boys don’t cry (or kiss or dance): A computational linguistic lens into gendered actions in film
Source: PLoS One. 2022 Dec 21;17(12):e0278604. doi: 10.1371/journal.pone.0278604 (PMC9770346; doi:10.1371/journal.pone.0278604)
Supplement: Attachment — Submitted filename: Response to reviewers.pdf [file pone.0278604.s006.pdf]

General Response

We thank the reviewers for their thoughtful feedback. We are encouraged by their recognition that the topic is “essential for organization’s effective and socially responsible communication” (R2) and that they found the manuscript and experiments well-written and sound with useful results (R1, R2).

A common concern among the reviewers was that the appendices contained valuable information that should have been included as part of the main body (R1, R2). We have since summarized the appendices into the paper itself, with a particular focus on the manual annotation process (as suggested by R1). Furthermore, we have followed R2’s suggestion on including a flow diagram in the introduction that we hope better explains the whole data processing and analysis stage.

Furthermore we have followed the style and grammar suggestions provided by the reviewers. In the following, we try to address the remainder of the reviewers points.

Response to Reviewer 1:

Q1: The main design issue with this research is related to the binary classification of gender, which is addressed in a note: "[...] we are limited by the content analysis procedures". This should be more developed and detailed in the limitations and perspectives, so that somebody who would like to do this is a more continuum-oriented way know what to do.

Author’s response: We deeply appreciate the reviewer’s comment, however at this time we have our reservations preventing us from venturing any suggestion on what a holistic continuum solution would even look like. While one possibility is to argue that we could represent a character’s expressed gender as a continuum variable, this is still limited by its inherent reliance on a (hetero-)normative onthology that places female–male as polar opposites. We believe that there is still a long-way to go in discussions and implementations before any automatic content analysis procedure can fully capture the nuances and inter-complexity of a character’s (and ultimately a person’s) identity in any comprehensive manner. We believe that these discussions, while still important to have, are out of the scope of our current work.

Q2: I'm also concerned by the variables being maybe not independent of others which are not taken into account here, like the socio-economic status of the characters. This is highlighted in the limitations concerning race and age, but not the socio-economic status.

Author’s response: There might be many hidden factors yet to be considered as explanations for action frequency, yet many of these are unattainable due to a lack of data. Furthermore, since our analysis is performed over a large sample of action descriptions, over a span of 4 decades, and controlling for the factors we do have data for, we suspect that these hidden relationships are marginalized in the aggregative results.

Q3: The experiment implies a number of layers, it is difficult to assess the quality of the intermediary results (of Spacy, for example). I suggest to add a table showing the different layers and the performance for each of them, if space allows.

Author's response: Our model relies on Spacy only to find the verbs inside the action descriptions. Spacy provides public performance benchmarks on their website (https://spacy.io/usage/facts-figures). Given that we have no reason to believe that the performance of our system would be greatly impacted had we used a different verb identification system, we decided not to include these. We do provide extensive comparisons against other SRL systems (with and without Spacy) in Table S1.

Q4: Figure 2 is unreadable to me: what is the meaning of the colors? where is the information about female/male characters? Also, in the huge tables in the Appendix, the best results should be in bold.

Author's response: We agree that Figure 2 failed to communicate our original intent and confused the discussion. We have since removed Figure 2 from the updated manuscript. We have also updated the table in the appendix to highlight the best model's performance in bold.

Q5: Some parts of the paper are a bit too optimistic and should be more nuanced. In particular, Table 2 results are described in the caption as " high precision and recall.", with a recall ranging from 0.63 to 0.8 (precision over 0.9). I suggest to correct this, in order to reflect the results, as the recall cannot be considered "high" here.

Author's response: We have updated the language used throughout the manuscript in an effort to avoid sounding overly optimistic.

Q6: The Appendix is longer than the paper itself and contains a lot of interesting information, some of which should appear in the paper itself, in particular concerning the manual annotation process. Concerning the usage of MTurk, the appendix states that the annotators received at least an hourly US minimum wage. Can you explain how and when it was ensured/computed? Which minimum wage (the (very low) federal one?)? Can you also state how many workers (if any) were rejected and for which reason?

Author's response: We have re-structured the manuscript to reflect your suggestions. We have now incorporated the information on dataset creation, methods and experiment (previously found in the appendix) as part of the main body.

We set the remuneration scheme for our task to ensure annotators receive at least minimum wage for their effort. This was calculated for the U.S. as $7.25/hr divided by the expected time it

would take to complete a single annotation. Before submitting our task, we did a few dry runs where we estimated the time required to complete a single annotation task. The labeling task (i.e., given an action description, selecting an agent and a patient from a dropdown box) took us about a minute per sample, while the verification task (i.e., deciding whether a proposed label is correct) took half a minute per sample. We have incorporated this response as part of the footnote in page 5.

With respect to the rejection rate, we found that some of the annotators submitted empty results (i.e., did not select a character from the drop-down). We marked their annotations as moot and rejected their tasks. This totalled to 2,162 rejected samples. Additional information can be found in lines 416–423.

Q7: "more feminine language": can you be more precise? What does "feminine language" mean in this paper?

Author's response: In this context we were referring to gender ladenness. Gender Ladenness, as defined in (Clark and Paivio, 2004) represents the degree of perceived "feminine or masculine association" on a numerical scale ranging from very masculine to very feminine. We have updated the manuscript to make the language clearer.

James M Clark and Allan Paivio. 2004. Extensions of the Paivio, Yuille, and Madigan (1968) norms. *Be- havior Research Methods, Instruments, & Comput- ers*, 36(3):371–383.

Q8: Legal issue: the raw corpus is not freely available, instead it is only available for "fair use". This should be cleared in the paper.

Author's response: We appreciate the reviewer's concern on the legality status of our work, however, assuming that *raw corpus* refers to ScriptBase, we can assure the reviewer that our use falls well within the limits established by the U.S. Copyright Law section 107 (fair use under non-commercial education and research purposes). If our assumption on what the reviewer meant is wrong, we will be happy to revisit this point.

Q9: I don't think the term "MWE" can be used for expressions like "Elron and Galdalf". These can be considered as named entities, which you do not want to split, but probably not as MWE. Criteria for MWE in UD can be found here:
https://universaldependencies.org/workgroups/mwe.html. More details:
https://hal.archives-ouvertes.fr/hal-03016721/document

Author’s response: The reviewer is correct in pointing this out. We have updated the manuscript to use the more appropriate term of *conjunction*.

Q10: Justification for the answer NO to "Have the authors made all data underlying the findings in their manuscript fully available?": the details of the results are available in the supplementary materials, but not the manually annotated data and the status of the dataset is not specified in the paper. This should be made clearer.

Author’s response: We have made all the annotated data and trained models available for anyone to download and use at https://sail/usc/edu/~ccmi/actions-agents-and-patients (Line 109–111).

Q11: Finally, 4 references are in fact pre-prints, not peer-reviewed, papers. I suggest to clearly state that they are preprints in the bibliography and to name them "technical reports".

Author’s response: Thank you for pointing this out. We have updated the manuscript to reflect that these are not peer-reviewed publications.

Response to Reviewer 2:

Q1: A flow diagram depicting the stages of data collection/ processing stages and analyses step might be handy to catch the reader’s attention and make the readers understand the whole data processing and analysis stage.

Author’s response: We thank the reviewer for this suggestion. We have updated the manuscript by including the suggested diagram as Fig 1.

Q2: The subtitles under the Conclusion section could be revised by considering the compatibility with the findings in general (For instance, 4.2 (The male-gaze theory" does not seem to be the compatible title. The authors use this base or approach just to explain their findings.)

Author’s response:

Q3: All of the information in the Appendices are valuable. However, this section seems to be too long as an Appendix. I suggest the authors summarize the quotes from "Materials" to "Experiments" and integrate this information into the main body of the MS. Some of the sections

in the Appendices, excluding Tables, are too long and detailed. The authors can put appropriate subtitles If they prefer or necessary. Furthermore, the flow diagram would visually complete the meaning of the whole process.

Author's response: We have re-structured the manuscript to reflect your suggestions. We have now incorporated the information on dataset creation, methods and experiment (previously found in the appendix) as part of the main body.

Q4: the authors state neglecting a time-trend factor in the models as one of the limitations of the study. Unfortunately, this might be an important analytical weakness rather than limitation.At this stage, I would like to ask why do not consider time series based analysis ?

Author's response: The reason behind not considering a time-trend as a factor were two fold. First, we did not have complete information on the years of production. Second, as stated, our original models did not converge due to technical limitations of the models–mainly that since they were developed using an old statistical package (based on R) it did not allow for parallel processing. We are happy to let the reviewers know that we have worked out these limitations by collecting additional year-of-release information, and re-writing our statistical models using the more modern Julia programming language. This allowed us to include an additional study (Study 4: Agent & Patients + Time) in which we include a movie's year-of-release as part of the control variables. The idea behind it is that this model captures the notion that different years have different film trends resulting in different action-frequency distributions. We have updated the manuscript incorporating the details on this time-based study (lines 404–412) and its respective results (lines 503–517) as well as updated the discussion to reflect our gained knowledge on how certain actions are significant even when controlling for yearly trends.
