# Supplementary material for: Boys don’t cry (or kiss or dance): A computational linguistic lens into gendered actions in film
Source: PLoS One. 2022 Dec 21;17(12):e0278604. doi: 10.1371/journal.pone.0278604 (PMC9770346; doi:10.1371/journal.pone.0278604)
Supplement: Attachment — Submitted filename: PLOS Response to reviewers 2.pdf [file pone.0278604.s007.pdf]

November 12th, 2022
PLOS One Editorial Team,

Dear Editorial Team,

We would like to thank the reviewer for their effort in providing insightful feedback to improve our work. Throughout this process, we continue to be encouraged by the reviewer's recognition of the interesting topic, and the value that our work provides to the discussion on gender bias in the media.

While working on this revision, we have identified and addressed a limitation in our statistical analysis that resulted in an overestimation of the number of significant coefficients. In summary, our Z-tests for coefficient significance (H0: \beta = 0 vs. H1: \beta != 0) were not controlled for multiple comparison errors (also known as familywise errors). To address this limitation, we incorporated a post-hoc Holm-Bonferroni correction for all GLME models. In other words, we are employing a higher standard for what we consider to be a significant result. The presented revision collects only the results that are found to be significant after correction (i.e., adj-p < 0.05).

We have updated the results, tables and discussion section to contextualize only those results that meet the new (and more appropriate) standard. This resulted in the removal of certain citations, as these are no longer needed.

Response to Reviewer 3:

Comment regarding the tables 5 to 8: We agree with the reviewers comment that the tables are hard to parse and not easy to understand. In addition to increasing the standard for what we consider a significant coefficient, we improve the readability of the tables in the supplementary materials as follows:

- We removed the significance column. This column aimed to provide an easy way to discern the power of the test (i.e., how likely is this coefficient to be different from zero?). However, after correcting for multiple comparisons, the coefficients deemed significant were those with the smallest p-values (most of which had adj-p = 0) which resulted in all having the same significance level.

- We followed R3's suggestion on presenting the coefficients ordered by their magnitude.

- Additionally, we followed R3's suggestion to split the tables into sub-tables according to the direction of the relationship (ie. less or more likely to be portrayed by X gender). This direction is given by the coefficient sign. We caption sub-tables with a brief explanation of the interpretation for each of the results presented (e.g., "Actions where the agent is more likely to be male").

Figure 1: We updated the figure to fix the layout issue. (Thanks for pointing this out).

Verification steps of the annotations: We have included a paragraph with a brief explanation of why we decided on a two-step verification process. In summary, we had to be certain that the labels met a minimum quality standard since the models we are using make no distinction between subjects (i.e., person, animal or thing that is in the receiving end of the action) and patients (ie. characters receiving actions).

In addition to the above comments, we have corrected additional spelling and grammatical errors.

We look forward to hearing from you in due time regarding our submission and to respond to any further questions or comments you may have.

Sincerely,

Dr. Victor R Martinez
Corresponding Author
